# Scope, Characteristics, Behavior Change Techniques, and Quality of Conversational Agents for Mental Health and Well-Being: Systematic Assessment of Apps

**DOI:** 10.2196/45984

**Published:** 2023-07-18

**Authors:** Xiaowen Lin, Laura Martinengo, Ahmad Ishqi Jabir, Andy Hau Yan Ho, Josip Car, Rifat Atun, Lorainne Tudor Car

**Affiliations:** 1 Lee Kong Chian School of Medicine Nanyang Technological University Singapore Singapore Singapore; 2 Psychology Programme, School of Social Sciences Nanyang Technological University Singapore Singapore Singapore; 3 Centre for Population Health Sciences, Lee Kong Chian School of Medicine Nanyang Technological University Singapore Singapore Singapore; 4 Palliative Care Centre for Excellence in Research and Education Singapore Singapore; 5 Department of Primary Care and Public Health, School of Public Health Imperial College London London United Kingdom; 6 Department of Global Health and Population, Harvard T.H. Chan School of Public Health Harvard University Cambridge, MA United States

**Keywords:** conversational agent, chatbot, mental health, mobile health, mHealth, behavior change, apps, Mobile Application Rating Scale, MARS, mobile phone

## Abstract

**Background:**

Mental disorders cause substantial health-related burden worldwide. Mobile health interventions are increasingly being used to promote mental health and well-being, as they could improve access to treatment and reduce associated costs. Behavior change is an important feature of interventions aimed at improving mental health and well-being. There is a need to discern the active components that can promote behavior change in such interventions and ultimately improve users’ mental health.

**Objective:**

This study systematically identified mental health conversational agents (CAs) currently available in app stores and assessed the behavior change techniques (BCTs) used. We further described their main features, technical aspects, and quality in terms of engagement, functionality, esthetics, and information using the Mobile Application Rating Scale.

**Methods:**

The search, selection, and assessment of apps were adapted from a systematic review methodology and included a search, 2 rounds of selection, and an evaluation following predefined criteria. We conducted a systematic app search of Apple’s App Store and Google Play using 42matters. Apps with CAs in English that uploaded or updated from January 2020 and provided interventions aimed at improving mental health and well-being and the assessment or management of mental disorders were tested by at least 2 reviewers. The BCT taxonomy v1, a comprehensive list of 93 BCTs, was used to identify the specific behavior change components in CAs.

**Results:**

We found 18 app-based mental health CAs. Most CAs had <1000 user ratings on both app stores (12/18, 67%) and targeted several conditions such as stress, anxiety, and depression (13/18, 72%). All CAs addressed >1 mental disorder. Most CAs (14/18, 78%) used cognitive behavioral therapy (CBT). Half (9/18, 50%) of the CAs identified were rule based (ie, only offered predetermined answers) and the other half (9/18, 50%) were artificial intelligence enhanced (ie, included open-ended questions). CAs used 48 different BCTs and included on average 15 (SD 8.77; range 4-30) BCTs. The most common BCTs were 3.3 “Social support (emotional),” 4.1 “Instructions for how to perform a behavior,” 11.2 “Reduce negative emotions,” and 6.1 “Demonstration of the behavior.” One-third (5/14, 36%) of the CAs claiming to be CBT based did not include core CBT concepts.

**Conclusions:**

Mental health CAs mostly targeted various mental health issues such as stress, anxiety, and depression, reflecting a broad intervention focus. The most common BCTs identified serve to promote the self-management of mental disorders with few therapeutic elements. CA developers should consider the quality of information, user confidentiality, access, and emergency management when designing mental health CAs. Future research should assess the role of artificial intelligence in promoting behavior change within CAs and determine the choice of BCTs in evidence-based psychotherapies to enable systematic, consistent, and transparent development and evaluation of effective digital mental health interventions.

## Introduction

### Background

Mental disorders affect approximately 18% of the global population [1-3], cause substantial health-related burden worldwide [4], and account for the largest burden of disease as measured by years lived with disability [5]. Despite the recognition of mental health disorders as a substantial cause of disease burden and demand for health care services [1,6,7], major treatment gaps remain [8,9]. Substantial barriers, such as the lack of access to professionals and resources [10] and negative perceptions and stigma [11], hinder help-seeking behaviors. Digital health interventions such as mobile apps and conversational agents (CAs) can help reduce these gaps [6] by improving access to care [12] and reducing treatment costs [13].

CAs are computer programs designed to mimic human conversations [14]. They include both rule-based scripted agents with constrained user input and responses and artificial intelligence (AI)–enhanced agents with the ability to respond to natural language input. CAs can communicate with individuals through text, speech, videos, or other methods of information exchange and can be embedded within social media platforms, websites, or smartphone apps [15-17]. With the increasing use of smartphones [18], there is potential for apps to reach wide audiences in a cost-effective and unobtrusive manner. In mental health settings, CAs can provide full-time, internet-based support for users seeking to manage mental disorders or improve their general well-being [15,16].

CAs often adopt a behavior change approach for mental health promotion, with cognitive behavioral therapy (CBT) [19] and behavioral activation [20] forming the basis of their interventions. These interventions are often complex and encompass several different activities [21-23] that are usually described with little detail about the active ingredients of the activities responsible for the improved health outcomes [24]. To improve the understanding and comparability of complex interventions, it is useful to standardize the identification and characterization of the active components that lead to the desired behavior outcomes [24]. Behavior change techniques (BCTs) are the smallest “observable and replicable components designed to change behavior,” which can be used alone or in combination with other BCTs [25]. Several classification systems have been developed to categorize BCTs, of which one of the most used is the BCT taxonomy v1 [24], which is a structured and comprehensive list of 93 BCTs. This taxonomy has been used in a range of health interventions such as smoking cessation, physical activity, healthy eating, and mental health [26-31].

Although there has been an increasing number of smartphone apps involving CAs for mental health [12], the capacity and range of these interventions in promoting behavioral change remain underexplored. Existing reviews analyzing apps for behavior change mostly focus on apps promoting physical health [32], mental health in specific populations [29], or the use of persuasive strategies [33]. Although these apps deliver health information and therapy-based exercises to users, they often do not deliver content using a conversational format that actively engages in a dialog with users, as seen in CAs. None of the reviews focused specifically on the use of CAs for mental health and well-being.

Therefore, to provide an overview and guidance for the development of future effective interventions [34], there is a need to characterize existing CAs and identify the types of BCTs they use.

### Objectives

This study aimed to systematically assess mental health and well-being CAs on Apple’s App Store and Google Play using the BCT taxonomy v1 [24]. Specifically, we aimed to do the following:

1. Identify the main characteristics of mental health CAs
2. Describe BCTs in the existing mental health CAs
3. Examine the use of chosen BCTs in relation to the CA characteristics and types of mental health interventions delivered by the CAs
4. Assess the technical aspects and quality of mental health CAs in terms of engagement, functionality, esthetics, and information using a validated scale

## Methods

### Overview

The search, selection, and assessment of apps used an established methodology [35-40]. The process involved a systematic search, 2 rounds of selection, and an evaluation following a predefined list of criteria adapted from previous studies [38-40]. The protocol was registered on the Open Science Framework [41] before data collection.

### Assessment Criteria

Information was extracted from the app store descriptions, app websites, and after testing the apps. The apps were evaluated based on the following assessment criteria that included four sections:

1. *General characteristics of the app and CA*, as described on the app store product page, were evaluated, including the developer’s name, app store category, version number, user rating, cost (if any), number of downloads, country of origin, and target users. The features of the CAs [14] comprised the personality traits of the CA, defined as personality codes of the CAs as described in individual studies, and other features such as the level of intelligence and duration of the user-CA relationship [42], defined as short term if the CA immediately responded to user queries in 1 or a few interactions and long term if the CA repeatedly interacted with the user over several interdependent sessions.
2. *Evidence-based mental health interventions delivered by CAs*, based on established clinical guidelines [43], a CBT competencies framework [44], a CBT manual [45], and components of existing CBT and third-wave therapies [19,46,47], were assessed. Apps designed for multiple mental disorders or no specific disorder were defined as “transdiagnostic” [48], as opposed to apps targeting a single specific mental disorder, such as reSET for substance use disorder [49]. The details of the assessment criteria are presented in Multimedia Appendix 1.
3. *Behavioral change techniques offered by the CA*, based on the BCT taxonomy v1 [24] that describes 93 distinct techniques arranged within 16 clusters, such as goals and planning, social support, reward and threat, antecedents, self-belief, and covert learning, were evaluated.
4. *Technical aspects and quality assessment of the app*, based on the Health on the Net Foundation certification of mobile applications (mHONcode) [50] and Mobile Application Rating Scale (MARS) [51,52], were assessed. The mHONcode evaluates credibility, safety, confidentiality, justifiability, ease of use, financial disclosure, and the use of advertising in apps. The MARS provides app quality and content rating scales that include engagement, functionality, esthetics, and information subscales as well as a subjective quality subscale based on researchers’ appraisal of the app. Items are rated on a Likert scale ranging from 1 (inadequate) to 5 (excellent). In line with the tool development paper [51], the total MARS score was calculated as the average of the subscales excluding the subjective quality.

### App Selection

A systematic search of the commercially available apps on Apple’s App Store and Google Play was performed on March 28, 2022, using 42matters [53], a mobile app market database. We searched the “Health and Fitness,” “Lifestyle,” and “Medical” app store categories using a search strategy comprising 24 search terms (Multimedia Appendix 2). A complementary Google search using the term *mental health chatbot* was also performed on April 29, 2022, and apps that were identified in the first 3 pages of the search results were added to enhance the rigor of the search. The inclusion and exclusion criteria for the apps are specified in Table 1.

**Table 1:** Inclusion and exclusion criteria of apps.

Criteria	Inclusion criteria	Exclusion criteria
Type of app	Free or paid apps available on Apple’s App Store or Google Play	Apps found only in the app stores not listed in the inclusion criteria
Language	Apps in English	Apps in languages other than English
Last update	Apps uploaded or updated from January 2020 onward	Apps uploaded or updated before January 2020
Scope of app	Apps that provided mental health care such as mental well-being promotion, assessment, or the management of mental disorders, including, but not limited to, depression, anxiety disorders, posttraumatic stress disorder, and substance use disorders	Apps that did not provide mental health care as specified in the inclusion criteria
CA^a^ component	Apps with a CA either stand-alone or as a component of a multifunctional app	Apps without any CA component
Technical issues and access	Apps without technical or access issues as specified in the exclusion criteria	Apps that could not be used after 2 attempts because of technical problems or required an access code provided by a third party (eg, insurance company or health care institution)
User ratings	Apps with >10 user ratings on the app store’s page	Apps with <10 user ratings on the app store’s page

^a^CA: conversational agent.

### App Assessment

**Figure 1:**
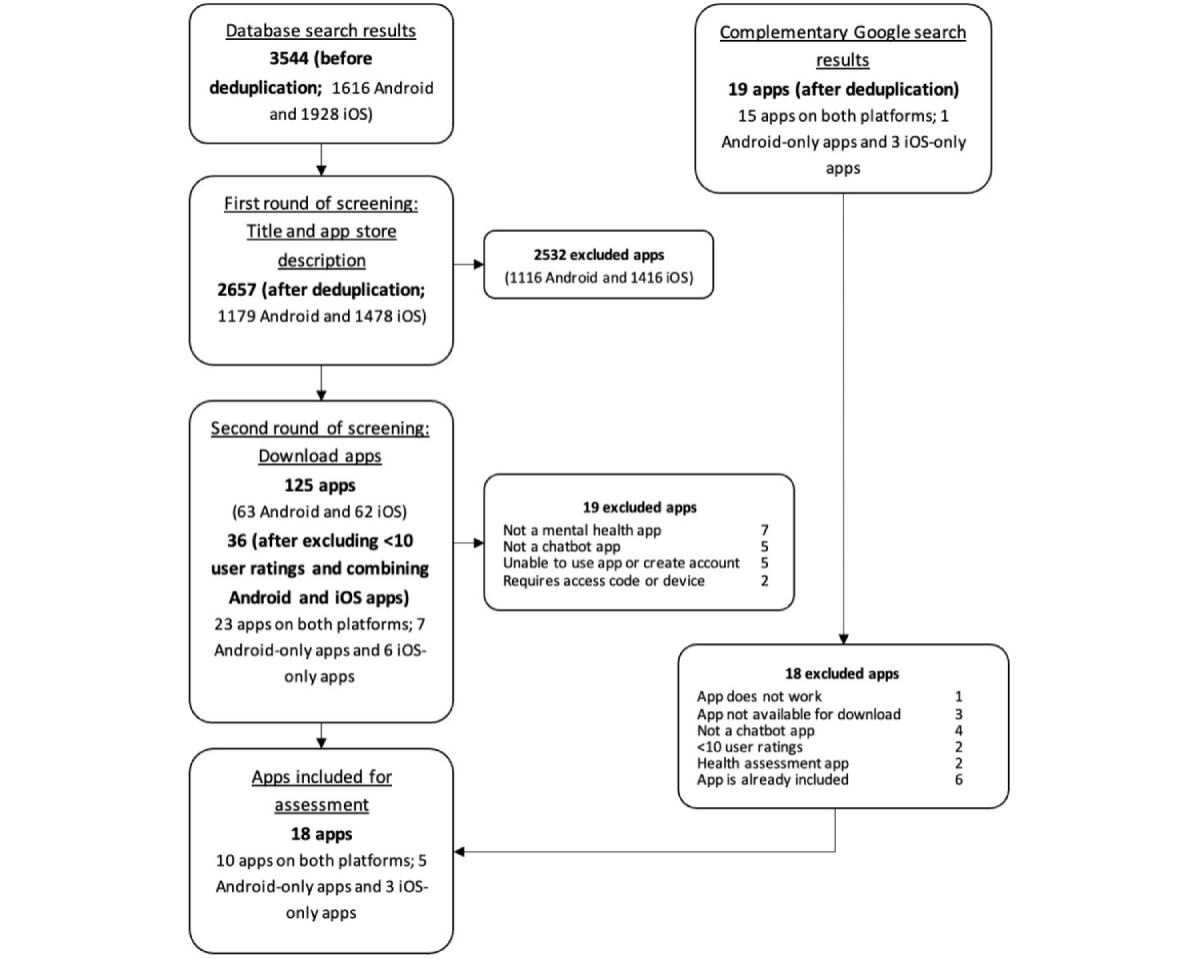
App selection flowchart.

The app selection process is illustrated in a flowchart (Figure 1). Two independent reviewers (LM and XL) worked in parallel to screen the app names and app store descriptions based on the eligibility criteria. After the first stage of screening, apps with <10 user ratings were excluded and those that were available on both app stores were combined. The remaining apps were downloaded onto smartphones and further assessed for eligibility in the second stage of screening. Any disagreements were resolved through consensus or discussion with a third reviewer (LTC).

Apps were downloaded and assessed using an iPhone 13 (iOS 15.4.1), Samsung A52 (Android 12, One UI 4.1), and Xiaomi Mi Max (Android 6.0.1) by at least 2 reviewers, including XL, LM, and 1 of 3 medical students who assisted with the project. If the apps were available on both platforms, both versions of the app were assessed and counted as 1 individual app. The data were extracted and tabulated using Microsoft Excel (version 2205; Microsoft Corp).

To ensure consistency and comprehensiveness of the assessment, the research team created 3 user personas with information relating to mental health, including the user’s name, demographics, medical history, opening statements with CAs, and answers to common self-reported questionnaires (ie, Patient Health Questionnaire 9 and Generalized Anxiety Disorder-7). For example, 1 profile was of a user with moderate depression that included symptoms of sadness, insomnia, and anhedonia, with the user staying at home most of the time and not looking for a new job. These personas were used by the study team to select or generate user responses when interacting with the CA. The web-based BCT taxonomy training program [54] was completed by the reviewers before data extraction to ensure reliability in the categorization of the BCTs. Apps were assessed for the available BCTs through repeated testing and the completion of modules within the app. BCTs were coded only when they were delivered directly by the CA through dialog or within an app component suggested by the CA. Each reviewer assessed the MARS scores for the apps, and the final result was defined by calculating the mean of both reviewers’ assessments. For scores that differed by >2 points on the Likert scale, indicating disagreement between reviewer assessments, the final result was defined by consensus between reviewers or in consultation with a third reviewer acting as an arbiter. The duration of app testing depended on the complexity of the content and modules offered by the CAs. In multifunctional apps, only the CAs were assessed in accordance with the objectives of this study.

### Data Analysis

Descriptive statistics were used to analyze the CAs’ characteristics, evidence-based mental health features, number of BCTs, app store user ratings, and MARS ratings using Microsoft Excel. BCTs that were frequently observed in >75% of apps in each subgroup were included in the narrative analysis, following the definitions of common BCTs that ranged from 50% to 80% in previous app reviews [29,32,55]. The number of user ratings was also used to examine the quality of the apps, as it was found to be related to the app’s degree of success [56].

For the classification of mental health and well-being topics, a free-for-use text analytics platform [57] was used to generate a word cloud from the app descriptions. For the apps available on both iOS and Android platforms, as the app descriptions were similar, only 1 description was included to avoid double counting of words. The top 50 frequently occurring words or phrases from the descriptions were included in the word cloud (Multimedia Appendix 3). The size of words indicated the relative frequency of occurrence after considering valid terms that can be found within other terms (eg, the term “mental health” is not counted with “good mental health”).

## Results

### App Search

The app search yielded 2657 apps (1616 from Android and 1928 from iOS) after removing duplicates (Figure 1). After the screening and excluding apps with <10 user ratings, 37 apps were downloaded for the second round of screening. A total of 18 apps were included in the assessment, comprising 5 Android-only apps, 3 iOS-only apps, and 10 apps available on both platforms (Multimedia Appendix 4).

### App Characteristics

More than half (10/18, 56%) of the included apps were available on both Android and iOS platforms (Table 2). The average app store rating was 4.25 (SD 0.74), ranging from 2.04 to 5. Two-thirds (12/18, 67%) of the apps had <1000 user ratings on both app stores. Most apps (15/18, 83%) were freely available for download, of which some apps followed a “freemium” model (5/15, 33%) that offered in-app purchases for additional content (Happify), analysis of user input (InnerHour), or premium courses that were not delivered by a CA (ie, GritX, IWill Care, and Zifcare) and were not assessed in this study. All included apps targeted people with mental disorders or the general population. None of the included CAs targeted health care professionals or caregivers.

**Table 2:** General characteristics of included apps (N=18).

Characteristic		Value, n (%)
**Number of downloads (Android)**		
	<100	1 (6)
	>1000	5 (28)
	>10,000	2 (11)
	>100,000	5 (28)
	>1,000,000	2 (11)
	N/A^a^	3 (17)
**Number of user ratings^b^**		
	<50	4 (22)
	50-100	3 (17)
	100-1000	5 (28)
	1000-5000	2 (11)
	>5000	4 (22)
**Pricing model**		
	Free	10 (56)
	Freemium	5 (28)
	Paid	3 (17)
**Minimum allowed age group (years)**		
	Young adult (>18)	9 (50)
	Adolescent (>13)	6 (33)
	No minimum age stated	3 (17)
**Input modality^c^**		
	Text	17 (94)
	Voice	3 (17)
	Emojis	2 (11)
	Images	2 (11)
**Output modality^c^**		
	Text	18 (100)
	Voice	7 (39)
	Emojis	9 (50)
	Images	7 (39)
	Video or Graphics Interchange Format	9 (50)
**CA^d^ personality^c^**		
	Coach like	15 (83)
	Informal	12 (67)
	Factual	11 (61)
	CA identity	10 (56)
	Knowledgeable	5 (28)
	Health care professional like	5 (28)
	Gender specific	2 (11)
	Human like	2 (11)
**Mental health and well-being topics^c^**		
	General well-being	18 (100)
	Stress	15 (83)
	Anxiety	13 (72)
	Depression	12 (67)
	Others^e^	7 (39)
**Therapeutic approach^c^**		
	Cognitive behavioral therapy	14 (78)
	Positive psychology	4 (22)
	Dialectical behavioral therapy	3 (17)
	Acceptance and commitment therapy	1 (6)
	Gestalt therapy	1 (6)
	None specified	2 (11)

^a^N/A: not applicable.

^b^Number of user ratings from the Apple App Store and Google Play were summed up for apps that were available on both platforms.

^c^Some apps may present with >1 feature within the sections; therefore, some sections do not add up to 100%.

^d^CA: conversational agent.

^e^Others included addiction, pain management, grief, loneliness, anger management, sleep hygiene, and relationship management.

### CA Characteristics

Most CAs (12/18, 67%) were represented by a cartoon avatar, often representing an object or animal, whereas one-third (6/18, 33%) of the CAs had no visual representation. Half (9/18, 50%) of the CAs used a rule-based approach that delivered predetermined responses, whereas the other 9 CAs included AI algorithms that allowed free-text input from users. However, it was apparent that AI-enhanced CAs used rule-based dialogs for therapeutic delivery, although AI was primarily used for understanding user input, selecting conversation responses, and possibly for selecting the treatment approach but not delivering the therapeutic content, such as psychotherapeutic exercises or information about mental health. For example, in Talk to Poppy, although conversational starters with the CA varied from time to time, the delivery of health information by the CA remained fixed and did not change over multiple repeated interactions, suggesting a rule-based approach for the delivery of therapeutic content. Most CAs (13/18,72%) were also part of multifunctional apps with other components such as human support and additional course content delivered not through CAs but via articles, stories, and motivational quotes for users, to cite a few.

Most CAs were encouraging, motivating, and nurturing (ie, coach like; 15/18, 83%) or informal (12/18, 67%). Approximately half (10/18, 56%) of the CAs explicitly identified themselves as a CA and not a person. One gender-specific CA (Inwords) provided 9 different voice output options.

Most CAs (14/18, 78%) engaged in multiple interactions with users to support the completion of long-term goals such as self-management of mental health conditions [42]. Alternatively, the short-term CAs [42] (4/18, 22%) answered user queries but were not involved in the delivery of the therapeutic content over multiple interactions. Specifically, the Mindspa CA was designed to assist users in dealing with difficult emotions or situations in emergencies only, whereas the CAs in Jumping Minds, Magnify Wellness, and Zifcare provided general support to users through the delivery of stories, motivational quotes, and jokes. Magnify Wellness and Zifcare also provided general information on CBT and some mental disorders, respectively. Table 2 lists the specific characteristics of CAs included in the analysis.

### Evidence-Based Mental Health Intervention Features

All 18 CAs aimed to improve mental health and well-being, including 2 apps (2/18, 11%) that addressed only general well-being. Most CAs targeted ≥1 mental health issues such as stress (15/18, 83%), anxiety (13/18, 72%), and depression (12/18, 67%) and often focused on all 3 mental health topics (13/18, 72%). General wellness topics included suggestions to reduce negative emotions (11/18, 61%) and advice to improve the quality of sleep (5/18, 28%), encourage personal growth (4/18, 22%), and manage relationships (4/18, 22%). Most CAs mentioned using evidence-based therapies, mainly CBT (14/18, 78%). Conversely, 2 apps (ie, Jumping Minds and Aiki) did not specify the therapeutic approach or techniques offered by the app.

In general, CAs included an average of 3.33 (SD 1.69) therapeutic techniques. Most CAs (16/18, 89%) offered patient education about the symptoms, diagnosis, or treatment of mental health conditions. However, less than half (7/16, 44%) of the apps included information about specific mental disorders such as depression and anxiety disorders. Most CAs (13/18, 72%) also offered relaxation techniques such as breathing exercises, mindfulness, and muscle relaxation. Half of the apps included behavioral activation (9/18, 50%) and cognitive restructuring (9/18, 50%) techniques. However, only a few apps that offered cognitive restructuring (3/9, 33%) also included behavioral experiments to test the accuracy of users’ maladaptive thoughts or beliefs. None of the CAs used exposure techniques to challenge users to engage in anxiety-provoking activities. Apps also offered other behavioral strategies such as emotional agility exercises for emotional regulation (Woebot), scheduling worry time as part of CBT (InnerHour and Iona), and encouraging positive self-affirmations (Iona) as described by positive psychology. The other techniques offered by the apps included journaling (9/18, 50%), mood monitoring (7/18, 39%), and gratitude exercises (5/18, 28%). Two CAs (Happify and Magnify Wellness) included video games aimed at reducing users’ worry and stress (Table 2).

Approximately a fifth (4/18, 22%) of the apps included an in-app user community. In 1 app (ie, Jumping Minds), users could chat with the members of the peer support network either individually or as a group. Moreover, 2 apps offered group chats with other users (ie, InnerHour and Happify), and another app (ie, tomo) shared users’ completed tasks with a few peers to encourage users. Some apps (4/18, 22%) also provided access to health professionals within the country of origin of the app (ie, India for Lissun, InnerHour, and IWill Care and Romania for I’m Fine).

Few CAs included protocols to manage emergencies such as risk of suicide. In total, 2 CAs (2/18, 11%) were able to assess users at the risk of suicide by responding to user statements that included the words “suicide” or “suicidal thoughts.” Moreover, only approximately a third (5/18, 28%) of the apps included crisis helpline phone numbers limited to the country in which the app was developed or a limited number of countries. One app (Iona; 1/18, 6%) provided a comprehensive list of crisis helpline numbers for 93 countries and regions (Multimedia Appendix 5).

### The Use of BCTs in Mental Health CAs

**Figure 2:**
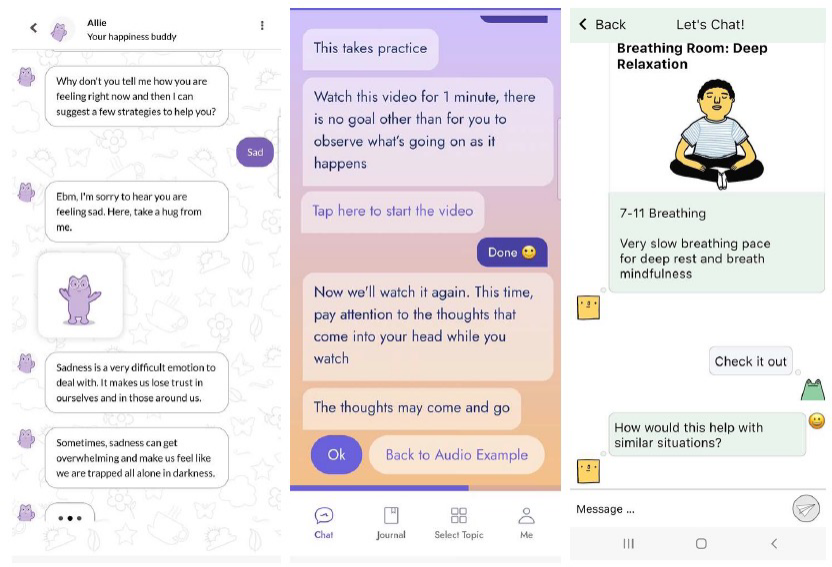
Screenshots of mental health conversational agents using behavior change techniques 3.3 (InnerHour or Amaha on the left), 4.1 (IonaMind in the middle) and 11.2 (GritX on the right).

On average, the apps included 15.8 (SD 8.77) BCTs, ranging from 4 (Zifcare) to 30 BCTs (Woebot; Multimedia Appendix 6). In total, 48 BCTs were found across the included apps (Multimedia Appendix 7). The most common BCTs included in >75% of apps were 4.1 “Instructions for how to perform a behaviour” (16/18, 89%), 3.3 “Social support (emotional)” (15/18, 83%), 11.2 “Reduce negative emotions” (15/18, 83%), and 6.1 “Demonstration of the behaviour” (14/18, 78%; Table 3). Figure 2 shows the presentation of some common BCTs in the selected apps. None of the apps used BCTs from category 14 “Scheduled consequences,” referring to scheduling rewards or punishments based on the performance of a behavior. The 4 short-term CAs commonly used 1 BCT, 3.3 “Social support (emotional).”

**Table 3:** Examples of how behavior change techniques (BCTs) were implemented among the included apps.

Common BCTs	BCT implementation
4.1 “Instructions for how to perform a behaviour”	“Watch this video for 1 minute, there is no goal other than for you to observe what’s going on as it happens... Now watch it again. This time, pay attention to the thoughts that come into your head while you watch” [Iona]
11.2 “Reduce negative emotions”	“Imagine yourself on some island where you feel safe and sound... Not only will this shift your mood, but it can also make you more productive at work when you get back to it.” [Aiki]
3.3 “Social support (emotional)”	“I’m sorry to hear you are feeling sad. Here, take a hug from me [GIF of CA^a^]” [InnerHour]
6.1 “Demonstration of the behaviour”	Describes another CA as someone with similar experiences of negative thoughts and how the CA overcame them (Talk to Poppy)
5.6 “Information about emotional consequences”	“This can help interrupt our negative thoughts and boost our experiences of pleasure, achievement, and mastery which can help change our mood” [Nuna]
12.6 “Body changes”	Relaxation exercises, for example, breathing space audio track and grounding (Wysa)

^a^CA: conversational agent.

### Choice of BCTs Across CAs, Mental Health Topics, Approaches, and Techniques

AI-enhanced CAs used a wider range of BCTs such as 5.6 “Information about emotional consequences” (8/9, 88%), 7.1 “Prompts/cues” (8/9, 89%), 1.4 “Action planning” (7/9, 78%), and 10.4 “Social reward” (7/9, 78%) compared with rule-based CAs.

We observed that BCT 12.6 “Body changes” was commonly used in CAs targeting anxiety (10/13, 77%) and was demonstrated through breathing and relaxation exercises. CAs aimed at depression treatment used 2 additional BCTs. These were 5.6 “Information about emotional consequences” (9/12, 75%) as a justification for engaging in the suggested activities for depression and 12.6 “Body changes” (9/12, 75%) through relaxation, breathing, and stretching exercises.

The use of BCTs in CAs also differed with respect to the therapeutic approach. CAs with CBT approaches commonly used 12.6 “Body changes” (12/14, 86%) and 5.6 “Information about emotional consequences” (11/14, 79%). CAs with mindfulness-based approaches also used the same additional BCTs 12.6 (8/10, 80%) and 5.6 (8/10, 80%). In these CAs, BCT 12.6 was used in physical activity and relaxation exercises, whereas BCT 5.6 was used to explain the benefits of the suggested tasks. CAs offering positive psychology approaches commonly included 6 additional BCTs, such as BCTs 5.1, 5.4, and 5.6 relating to information about health and emotional consequences; 8.1 “Behavioural practice or rehearsal”; 12.4 “Distraction”; and 12.6 “Body changes” (Multimedia Appendix 8).

### Technical Aspects and Quality Assessment of the Apps

Regarding the technical aspects, all CAs required a stable internet connection to function. Most apps (14/18, 78%) could send notifications to remind the users of scheduled activities and conversations with the CA, and most apps (12/18, 67%) also required users to sign up for an account. Only 1 CA (Aiki; 1/18, 6%) used phone sensors for additional data analytics by using the microphone to collect the user’s voice input for mood analysis.

For the evidence base, only a few apps (3/18, 17%) were previously evaluated in studies such as qualitative research (Wysa and Happify) and randomized trials (Woebot), whereas other apps (Nuna and tomo; 2/18, 11%) reported ongoing studies.

### mHONcode Qualifications

In general, apps showed high compliance with 5 (63%) out of the 8 mHONcode principles. Most apps clearly stated the qualifications of app development teams (14/18, 78%), provided disclaimers that the app does not replace health care providers’ advice (17/18, 94%), included the date of the last update (18/18, 100%), indicated funding sources or companies that commissioned the app (16/18, 89%), and had no advertisements (18/18, 100%). Notably, many apps (15/18, 83%) were either inconsistent or did not provide health references to the information included on the app or their websites. Finally, the majority of apps did not request for user consent before data collection (16/18, 89%) or check the use of the apps by minors (16/18, 89%; Multimedia Appendix 9).

### MARS Assessment

**Figure 3:**
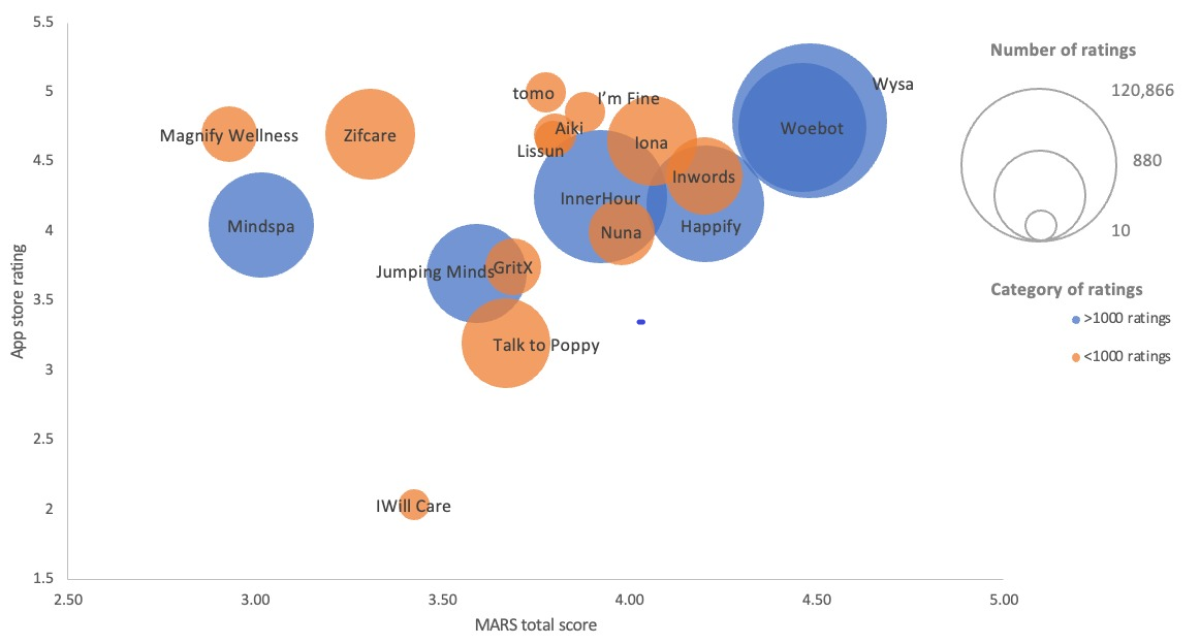
App store ratings and Mobile Application Rating Scale (MARS) scores of the included apps by the number of user ratings (N=18).

The average quality score of the included apps was 3.79 (SD 0.43), comprising engagement (mean 3.33, SD 0.67), functionality (mean 4.15, SD 0.47), esthetics (mean 4.12, SD 0.47), and information (mean 3.55, SD 0.52). The average MARS subjective score was 2.85 (SD 0.77), ranging from 1.38 to 4.38 (Multimedia Appendix 10). Figure 3 illustrates the app store ratings and total MARS scores for CAs categorized by the number of user ratings. For CAs with >1000 user ratings, the user app store ratings and total MARS score appeared to be positively correlated, with the exception of Mindspa.

## Discussion

### Principal Findings

This study systematically assessed the BCTs in 18 app-based mental health CAs in relation to their characteristics and quality. The CAs included 15 BCTs on average, of which the most commonly used were BCTs 4.1 “Instructions for how to perform a behaviour,” 3.3 “Social support (emotional),” 11.2 “Reduce negative emotions,” and 6.1 “Demonstration of the behaviour.” Most CAs used similar BCTs across different therapeutic interventions and topics. CAs that incorporated AI used a larger number of BCTs than purely rule-based CAs.

Of the common BCTs, only BCT 3.3 “Social support (emotional)” was frequently used in both short-term and long-term CAs. These findings suggest that the provision of social support may be one of the key functions of mental health CAs, as it was present even in short-term CAs that did not deliver therapeutic content over multiple sessions with the user. However, CAs that delivered interventions focusing on problem-solving or behavioral activation techniques did not offer emotional social support, but they offered other forms of support such as providing advice on where to seek social support and encouraging help seeking. This finding is in line with a previous review on the use of health apps for behavior change [58] and another review describing behavior change strategies to reduce sedentary behavior [59], in which unspecified social support was identified as one of the frequently used BCTs. In contrast, a recent systematic review evaluating the effectiveness of physical and mental health apps in improving health behaviors did not observe social support as a common BCT, arguing that more research is needed to evaluate the correct number and association of BCTs [31]. Other frequently observed BCTs in this assessment were BCTs 4.1 “Instructions for how to perform a behaviour” [29,31,32], 6.1 “Demonstration of the behaviour” [55], and 11.2 “Reduce negative emotions” [55], which parallels other studies on physical health– [29,31,32], mental health– [29,31], and sleep-related [55] apps. In our review, these BCTs were common across most therapeutic approaches and techniques. We suggest that these BCTs can typically be expected in mental health CAs, especially in longer-term CAs directly involved in the delivery of therapeutic content over multiple interactions with users. As most mental health apps aim to support an individual’s self-management [39], it is essential that they provide comprehensive information to educate individuals on the characteristics of mental disorders and the health care strategies better suited to improve mental well-being through BCTs 4.1 and 6.1. Further research that compares the use of BCTs in mental health care CAs is essential to improve our understanding of the mechanisms of behavior change associated with improved mental health outcomes.

Approximately one-third (5/14, 36%) of CAs claiming to be based on CBT did not include core CBT concepts such as behavioral activation, cognitive restructuring, and behavioral experiments [60,61]. For instance, none of the CBT-based CAs included BCT 1.4 “Action planning,” an essential component in behavioral activation tasks, or BCT 13.2 “Framing/Reframing,” associated with cognitive restructuring exercises. These findings are consistent with previous studies, in which mental health apps claiming to include CBT may not actually adhere to the core concepts of CBT [40,62,63]. Furthermore, the majority of the apps did not provide references to support the evidence basis of their health claims. This could imply that some app developers might be using scientific terminology to attract users [63]. In contrast, the lack of congruence with existing evidence may highlight the difficulty in translating traditional face-to-face therapies into mobile health (mHealth) interventions [12]. As such, a more granular analysis focusing on the use of BCTs using a standardized framework such as the BCT taxonomy v1 may allow a more accurate and transparent translation of the effective components in these complex interventions into mHealth interventions. However, although the use of BCTs in diet, physical activity, and smoking cessation interventions [26-28] has been well documented, there is a dearth of studies on the use of BCTs mapping to specific components of psychotherapy interventions. Further research on the systematic identification of BCTs in evidence-based therapies is needed for these techniques to be easily implemented in mHealth interventions for mental health.

A noteworthy finding was that AI-enhanced CAs included a greater variety of BCTs than rule-based CAs, an observation that has not been replicated in other similar studies. This finding suggests that the use of supervised machine learning and natural language processing techniques to predict, identify, and provide appropriate treatment options [64] may support the delivery of a more comprehensive therapeutic intervention. However, our assessment indicated that the delivery of therapeutic content appeared to be largely driven by rule-based programming rather than machine learning, which may have been used to ensure high fidelity in the delivery of the intervention. As errors in the delivery of health care interventions may have severe consequences for users [65], using fixed rule-based algorithms would ensure that the intervention follows an intended structure [66]. An alternative explanation could be that the teams involved in the development of AI-enhanced CAs had more resources to develop more sophisticated CAs that could deliver a larger variety of therapeutic components and BCTs. The advantage of including AI components may then be to personalize the intervention and the technique choices for each individual user. A more thorough investigation of the use of AI for specific components of CAs and in relation to the delivery of BCTs is warranted to gain better insight into the impact of using AI for behavior change.

Approximately two-thirds of the CAs in this assessment had <500,000 Android downloads and <1000 user ratings, and only 2 CAs had >1,000,000 Android downloads and >20,000 ratings on both app stores. This contrasts with earlier reviews of mHealth apps that had >50,000 user ratings on average [32,67,68]. In those reviews, few apps accounted for the bulk of the total downloads and ratings, whereas the remaining majority were much less used or rated. Similarly, in our review, we observed that the top 2 CAs accounted for 82% of the total user ratings and approximately 60% of the total downloads, which corresponds with a previous report on mHealth apps [18]. We identified 3 reasons that might account for the lower use of some of the mental health CAs. First, users might have concerns about the limitations of some mental health CAs in understanding their inputs and providing suitable dialog responses [69], as reported in previous studies on health care CAs [70,71]. This is also in line with the other limitations of the mental health CAs in this study, in which <30% of the CAs did not request for user consent before data collection, include health references for the information provided, check the use of apps by minors, or include protocols to manage emergencies such as suicide risk, raising concerns about the confidentiality, justifiability of information, users’ practice, use by minors, and emergency safety netting. As users might perceive the quality of conversations or the CAs themselves to be poorer than expected, this could reduce their likelihood of using certain mental health CAs compared with other mental health CAs or apps. Second, potential users might not be familiar with these mental health CAs and thus would not have engaged with CAs, as has been previously reported for mental health professionals [72]. Third, individuals could have different preferences for mHealth interventions, and some might choose not to engage with CAs, as they prefer features that are found in other health apps. These findings could be instructive for subsequent CA developers to consider the quality and justifiability of the information provided in conversations, the confidentiality of data, user access and appropriateness, and emergency management in the design of mental health CAs.

According to the MARS assessment, the average mental health CA was highly functional, easy to learn and navigate, visually esthetic, appealing, and stylistically consistent. In contrast, it was lacking in engagement and interactivity as well as the quality of the information provided. This finding is consistent with previous assessments of mindfulness [73] and health [32] apps. We also observed that for CAs with >1000 user ratings, the quality of apps as rated by the MARS appeared to be positively correlated with user ratings. In general, user ratings may not be a trustworthy indicator of an app’s quality, as positive reviews can easily be fabricated [74]. Previous studies also found no substantial correlations between user ratings and MARS scores in apps for mindfulness [73] and mental health [75]. Preliminary findings from our study, however, support that user ratings could potentially reflect the quality of apps that were more highly used and reviewed [56], although this finding was not supported in another study that compared user ratings and standardized expert rankings of health apps [76]. A more robust analysis is required to support this finding.

### Strengths and Limitations of the Study

We used a search strategy for app search, selection, and assessment that was based on a systematic review methodology. The apps were evaluated based on a comprehensive list of criteria and a reliable procedure for identifying the active components of health interventions using the BCT taxonomy v1. The use of the web-based BCT taxonomy training program before data extraction also ensured consistency in the specification of BCTs across reviewers.

However, this study has some limitations. First, although we aimed to retrieve all commercially available mental health apps with CAs through a systematic search strategy at the time of the search, owing to the expansive growth of digital health apps [18], relevant mental health apps may have been newly published and not fully captured in this analysis. Second, as apps were restricted to those available in the English language, apps published in other languages and countries may not have been well represented in this study. Third, we did not perform any statistical analyses as planned because of the small sample size of included apps. Finally, as this study only assessed CAs, the BCTs and MARS scores for the multifunctional apps may not reflect the assessment of all the other app components and therefore may differ from the assessments of the entire app.

### Conclusions

Most app-based mental health CAs were transdiagnostic and targeted several mental health issues such as stress, anxiety, and depression, which is reflective of a broad therapeutic focus. The provision of emotional support, instructions to perform a behavior, and strategies to reduce negative emotions were the most common BCTs identified in mental health CAs, supporting their role in the self-management of mental disorders. There were only a few highly used CAs, and most CAs were infrequently downloaded and used. Future CA developers should consider the quality of the information provided by CAs, user confidentiality, user access and appropriateness, and emergency management in the design of mental health CAs. There remains a need to clarify the role of AI in the provision of BCTs in CA-based interventions that target mental health promotion, and further research is required for the systematic identification of BCTs in evidence-based psychotherapies such that effective techniques can be effectively implemented in future mHealth interventions.

## Appendix

Multimedia Appendix 1 Details on the assessment criteria.

Multimedia Appendix 2 Search terms.

Multimedia Appendix 3 Word cloud of the app store descriptions for the 18 included conversational agents.

Multimedia Appendix 4 Characteristics of the included apps.

Multimedia Appendix 5 Other characteristics of the apps.

Multimedia Appendix 6 Number of behavior change techniques found in each app.

Multimedia Appendix 7 Number of conversational agents (N=18) using each behavior change technique.

Multimedia Appendix 8 Common behavior change techniques.

Multimedia Appendix 9 Health on the Net Foundation certification of mobile applications characteristics of the included apps.

Multimedia Appendix 10 Ratings of the included apps.

## Abbreviations

AI: artificial intelligence
BCT: behavior change technique
CA: conversational agent
CBT: cognitive behavioral therapy
MARS: Mobile Application Rating Scale
mHealth: mobile health
mHONcode: Health on the Net Foundation certification of mobile applications

## Data Availability

Data can be made available by emailing the corresponding author.
