# Supplementary material for: Scope, Characteristics, Behavior Change Techniques, and Quality of Conversational Agents for Mental Health and Well-Being: Systematic Assessment of Apps
Source: J Med Internet Res. 2023 Jul 18;25:e45984. doi: 10.2196/45984 (PMC10394504; doi:10.2196/45984)
Supplement: Multimedia Appendix 1 [file jmir_v25i1e45984_app1.docx]

**Multimedia Appendix 1.** Assessment criteria of the apps.

| Field | Description | Options | Additional Information |
| --- | --- | --- | --- |
| **1. App Description (from App Store)** | | |  |
| App ID | A unique ID number generated for each app for easy identification and merging of data. | Alpha-numeric |  |
| App Name | The given name/title of the app. | Free text |  |
| Developer name | The company of the app developer. If there is no name, note the developer’s name. | Free text |  |
| App store category | The category of the app in the app store | Health & Fitness/ Lifestyle/Medical |  |
| Platform | Is this app tested on Android or iOS? | Android/iOS |  |
| App version number | List the latest version of the app during assessment | Alpha-numeric |  |
| User rating | State user ratings if applicable on a scale of 5. | Numeric |  |
| Number of people who rated | State the number of people who provided ratings for the app at the point of assessment. | Numeric |  |
| Number of user downloads | State the number of times the app has been downloaded. | Numeric |  |
| Cost (App) | Cost to download the app (basic version). | Numeric |  |
| Cost (in-app purchases) | Cost for upgrade version of the app with extra features (if any). | Numeric |  |
| Accessible region | Is this app limited to certain locations/ countries/ regions? | Free text |  |
| Target user | Who is the target user of the app? | Adult Patient/ Caregiver/HCP/ others |  |
| Age group | What is the age group of target user? (Choose all that apply) | Children (under 12)/ Adolescents (13-17)/Young adults (18-25)/Adults/General |  |
| Topics on mental health and wellness | Which aspects of mental health does the chatbot target? (Choose all that apply) | Increase Happiness or Wellbeing /Mindfulness, Meditation, or Relaxation/ Reduce negative emotions/ Depression/Anxiety or Stress/Alcohol or Substance Use/Other (free text) | Includes general well-being, stress, depression, anxiety, and other topics obtained using word cloud frequency analysis |
| Intervention | What types of interventions are mentioned in the app? (Choose all that apply) | CBT - Behavioural (positive events)/ CBT – Cognitive (thought challenging)/ ACT - Acceptance commitment therapy /Mindfulness or Meditation/ Relaxation/ Gratitude/ Other (free text) |  |

**Supplementary Table 1.** Assessment criteria of the apps (continued).

| Affiliation | What kind of organisation is affiliated with this app? | Unknown/Commercial/ Government/NGO/University |  |
| --- | --- | --- | --- |
|  | Are professionals with relevant expertise (e.g., psychiatrist, psychologist, social worker for mental health) involved in the organisation? | Yes/No |  |
| Technical aspects of app | What technical aspects are included in the app? (Choose all that apply) | Allows sharing (Facebook, Twitter, etc.)/ Has an app community/ Allows password-protection/ Requires login/ Sends reminders/ Needs web access to function/Includes user profile/ Allows exporting of data |  |
| **CA specific information** | |  |  |
| Intelligence of CA | Is the CA app rule based or artificially intelligent? | Rule based/AI/Both | Rule-based: CA only used predefined keywords and commands  AI: CA could identify and respond with appropriate suggestions rather than predefined answers |
| Input modalities | Input all that apply | Predetermined Text/Free Text/ Voice/ Images/ Emojis/ Video |  |
| Output modalities | Input all that apply | Text/ Voice/ Images/ Emojis/GIFs/Video |  |
| Behaviour change | Does the CA target short term or long-term change of behaviour? | Short term (response or service almost instantaneously)/ Long term (needed to build a relationship with the user over time) |  |
| Default message | Is there a default message that pops up when the user enters information that the CA cannot understand or recognise? | Yes/No |  |
|  | What is the message? | Free text |  |
| Avatar | Is the CA embodied? | ECA/Non-ECA | ECAs are cartoon-like characters that present similarly to humans in face-to-face conversations. They can emulate and respond to verbal and non-verbal communication. |
|  | What does the avatar look like? | Free text |  |
|  | Can the user make changes to the appearance? | Yes/no |  |

**Supplementary Table 1.** Assessment criteria of the apps (continued).

| Personality | What is the personality of the CA. (options from our scoping review) | Coach-like, CA identity, culture specific, factual, gender specific, healthcare professional like, human-like, informal, knowledgeable | Coach like: Nurturing and motivating  Conversational agent identity: Identified explicitly as a CA Health care professional like: Mimics a doctor or expert Informal: Used exclamations, short-form, and emoticons in casual conversations Human like: Mimics human conversations Gender specific: Has male and female CAs Culture specific: Has local names or speaks the local language Knowledgeable: Written or informed by medical professionals Factual: Were objective and founded on facts or observations |
| --- | --- | --- | --- |
| Personalization | Does the app have any features which promote personalisation? | Yes/No | (e.g., Timings, record a history of user's preferences/health status, goals etc.) *Addressing user by name alone is not personalisation? |
|  | If yes, does it use personalised timings? | Yes/No |  |
|  | If yes, does it achieve personalisation by recording a history of the user's preferences? | Yes/No |  |
|  | If yes, does it record a history of the user's health status? | Free text (e.g., Symptoms, subjective health ratings, functional impairments, etc.) |  |
|  | If yes, does it keep a record of user's goals? | Yes/No |  |
|  | If yes, what other method of personalisation does the app employ? | Free text |  |
|  | Does the chatbot remembers previous conversations with the user? |  |  |
| CA integration | How integrated is the chatbot function within the app? | Standalone chatbot/Integrated with other functions |  |
| Health tracking | Does the chatbot provide any self-monitoring tracking functions (e.g., mood, behaviour, journaling, or others that require user input)? | Yes/No | E.g., mood, behaviour, thoughts, journalling, physiological markers, etc. |
|  | If yes, what types of active tracking functions are employed by the chatbot? (Choose all that apply) | Mood/Behaviour/Clinical Symptom/Others (specify) |  |

**Supplementary Table 1.** Assessment criteria of the apps (continued).

| Passive sensing | Does the app employ any passive sensing of user behaviour? | Yes/No |  |
| --- | --- | --- | --- |
|  | What types of passive sensing via physical sensors are employed by the chatbot? (Choose all that apply) | Location/Camera/Microphone/ Body Sensors/Physical Activity/Others (free text) |  |
|  | What types of passive sensing via phone usage information are employed by the chatbot? (Choose all that apply) | Call Logs/Text Messages/Screen Time/App Usage/Battery Level/Ambient Light/Others (free text) | Trifan et al., 2019: Passive Sensing of Health Outcomes Through Smartphones: Systematic Review of Current Solutions and Possible Limitations |
| Information & education | Does the app provide education/information about mental health conditions (e.g., symptoms, diagnosis, treatments, others)? | Yes/No |  |
|  | Does the app provide more specific information to educate the user on a particular mental health condition of focus? |  | Risk factors (psychological or environmental), safety plan, emergency management |
| Evidence | Is the information in the app backed by evidence? | Yes/No | Scientific paper, or a reputable website, e.g., NHS Health A to Z, Medline Plus, etc |
|  | If yes, include citation for the research paper(s) |  |  |
|  | Has the CA been evaluated in any research studies? | Yes/No |  |
|  | If yes, include citation for the research paper(s) |  |  |
|  | Have the developers published any scientific articles related to the design and development of the CA? | Yes/no |  |
|  | If yes, include citation for the research paper(s) |  |  |
| Human involvement | Does the app have a separate chat channel to allow the user to share information or contact people in her/his support network? | Yes/No | Related to interactions with other users, peers, or healthcare professionals in the app, e.g., family members, friends, support groups, others |
|  | Does the app include a health professional who is accessible to the user (without users inputting any health provider/ health professional information)? | Yes/No |  |
| Emergency safety netting | Does the app screen users for risk of suicide? | Yes/No | Referring to suicide risk assessment and access to emergency services provided by the CA |
|  | Does the CA allow the user to contact emergency services directly through the app? | Yes/No |  |

**Supplementary Table 1.** Assessment criteria of the apps (continued).

| **Use of BCTs by the CAs (go to "BCTs" sheet)** | | | |
| --- | --- | --- | --- |
| BC theories or frameworks used | Social Cognitive Theory/ Theory of Planned Behaviour/ Transtheoretical (stages of Change) Model/ Theory of Reasoned Action/ Health Belief Model/ Other (please specify) |  |  |
| Number of BCTs | Number of unique BCTs found | Numeric |  |
| **BCTs used in detail** | | | |
| 1. Goals & planning | Includes: goal setting, problem solving, action planning, review goals, commitment | Specify all BCTs from the group included by the CA |  |
| 2. Feedback and monitoring | Includes: Monitoring others or self, feedback on behaviour of others or self, biofeedback, | Specify all BCTs from the group included by the CA |  |
| 3. Social support | Includes: unspecified, practical or emotional support | Specify all BCTs from the group included by the CA |  |
| 4. Shaping knowledge | Includes: skills training, information about antecedents, re-attribution, behavioural experiments | Specify all BCTs from the group included by the CA |  |
| 5. Natural consequences | Includes: information about health, social and environmental, and emotional consequences, salience of consequences, monitoring of emotional consequences and anticipated regret | Specify all BCTs from the group included by the CA |  |
| 6. Comparison of behaviour | Includes: demonstration of behaviour, social comparison, and information about others approval | Specify all BCTs from the group included by the CA |  |
| 7. Associations | Includes: prompts & cues, cue signalling reward, reduce prompts & cues, remove access to reward, remove aversive stimulus, satiation, exposure, associative learning | Specify all BCTs from the group included by the CA |  |
| 8. Repetition and substitution | Includes: behavioural practice or rehearsal, behaviour substitution, habit formation and reversal, overcorrection, generalization of target behaviour, graded tasks | Specify all BCTs from the group included by the CA |  |
| 9. Comparison of outcomes | Includes: credible source, pros and cons, comparative imagining of future outcomes | Specify all BCTs from the group included by the CA |  |

**Supplementary Table 1.** Assessment criteria of the apps (continued).

| 10. Reward and threat | Includes: material incentive & reward, non-specific incentive & reward, social incentive & reward, self-incentive & self-reward, future punishment, incentive & reward for outcomes | Specify all BCTs from the group included by the CA |  |
| --- | --- | --- | --- |
| 11. Regulation | Includes: pharmacological support, reduce negative emotions, conserving mental resources, paradoxical instructions | Specify all BCTs from the group included by the CA |  |
| 12. Antecedents | Includes: restructuring the physical or social environment, avoidance, distraction, adding objects to environment, body changes | Specify all BCTs from the group included by the CA |  |
| 13. Identity | Includes: identification of self as role model, framing & reframing, incompatible beliefs, valued self-identity, identity associated with changed behaviour | Specify all BCTs from the group included by the CA |  |
| 14. Scheduled consequences | Includes: behaviour cost, punishment, remove reward or punishment, reward approximation, reward completion, situation-specific reward, reward incompatible or alternative behaviour, reduce reward frequency | Specify all BCTs from the group included by the CA |  |
| 15. Self-belief | Includes: verbal persuasion about capability, mental rehearsal of successful performance, focus on past success, self-talk | Specify all BCTs from the group included by the CA |  |
| 16. Covert learning | Includes: imaginary punishment, imaginary reward, vicarious consequences | Specify all BCTs from the group included by the CA |  |
| Remarks from reviewer |  | Free Text |  |
| **Therapeutic techniques used in detail** | |  |  |
| Patient Education | Does the app involve patient education (on symptoms, diagnosis, treatment principles, etc.?) | Yes/No | "Cognitive behaviour therapy is educative… the nature and course of her disorder, ..." (Beck) (** The CBT education component is… focused on ABC, i.e., teaching link between thoughts emotions and actions) |
| Behavioural Activation | Does the app guide users to overcome "depressive-passivity" and become more active through behavioural activation? (e.g., scheduling activities, tracking routine, pleasurable activity suggestions)? | Yes/No | "Behavioural activation is essential for most depressed patients. Many patients need only to be provided with a rationale, guidance in selecting and scheduling activities, and responses to predicted automatic thoughts that might interfere with implementing the activities or with gaining a sense of pleasure or mastery from them." (Beck) |

**Supplementary Table 1.** Assessment criteria of the apps (continued).

| Cognitive Restructuring | Does the app guide users through cognitive restructuring (e.g., identifying automatic thoughts and/or associated triggers and emotions, cognitive distortions, core beliefs)? | Yes/No | "Cognitive behaviour therapy teaches patients to identify, evaluate, and respond to their dysfunctional thoughts and beliefs." "Learning to evaluate automatic thoughts is a skill..." (Beck) |
| --- | --- | --- | --- |
| Behavioural Experiments | Does the app challenge users' thoughts using behavioural experiments? | Yes/No | "Therapists also create experiences, called behavioural experiments, for patients to directly test their thinking" (Beck) |
| Problem Solving | Does the app guide users to problem solve? | Yes/No | "Help alleviate their distress through a variety of techniques and problem solving." "Behavioural and problem-solving techniques are essential, as are techniques from other orientations that are implemented within a cognitive framework." (Beck) |
| Relaxation | Does the app suggest users to practice relaxation techniques (e.g., breathing, mindfulness, muscle relaxation)? | Yes/No | "Many patients benefit from learning relaxation techniques..." (Beck) |
| Exposure | Does the app challenge users to engage in activities that generate anxiety? | Yes/No | "When patients are anxious and significantly avoidant, you will provide a strong rationale for exposing themselves to feared situations" (Beck) |
| Positive psychology | Does the app involve positive psychology techniques (e.g., gratitude, savouring, practicing kindness, using personal strengths)? | Yes/No | "Positive Psychotherapy is an empirically supported approach to psychotherapy that attends specifically to building client strengths and positive emotions and increasing meaning in the lives of clients to alleviate psychopathology and foster happiness (Rashid, 2008; Seligman, Rashid, & Parks, 2006)." (Magyar-Moe et al., 2015) |
| Other modules | Does the app offer other functionalities to users? | Yes/No |  |
|  | If yes, what functionalities does the app offer? | Free text |  |
| **MARS App Quality Ratings** | |  |  |
| Engagement – fun, interesting, customisable, interactive (e.g., sends alerts, messages, reminders, feedback, enables sharing), well-targeted to audience | Entertainment: Is the app fun/entertaining to use? Does it use any strategies to increase engagement through entertainment (e.g., through gamification)? | 1 Dull, not fun or entertaining at all 2 Mostly boring 3 OK, fun enough to entertain user for a brief time (< 5 minutes) 4 Moderately fun and entertaining, would entertain user for some time (5-10 minutes total) 5 Highly entertaining and fun, would stimulate repeat use |  |

**Supplementary Table 1.** Assessment criteria of the apps (continued).

|  | Interest: Is the app interesting to use? Does it use any strategies to increase engagement by presenting its content in an interesting way? | 1 Not interesting at all 2 Mostly uninteresting 3 OK, neither interesting nor uninteresting; would engage user for a brief time (< 5 minutes) 4 Moderately interesting; would engage user for some time (5-10 minutes total) 5 Very interesting, would engage user in repeat use |  |
| --- | --- | --- | --- |
|  | Customisation: Does it provide/retain all necessary settings/preferences for apps features (e.g., sound, content, notifications, etc.)? | 1 Does not allow any customisation or requires setting to be input every time 2 Allows insufficient customisation limiting functions 3 Allows basic customisation to function adequately 4 Allows numerous options for customisation 5 Allows complete tailoring to the individual’s characteristics/preferences, retains all settings |  |
|  | Interactivity: Does it allow user input, provide feedback, contain prompts (reminders, sharing options, notifications, etc.)? Note: these functions need to be customisable and not overwhelming in order to be perfect. | 1 No interactive features and/or no response to user interaction 2 Insufficient interactivity, or feedback, or user input options, limiting functions 3 Basic interactive features to function adequately 4 Offers a variety of interactive features/feedback/user input options 5 Very high level of responsiveness through interactive features/feedback/user input options |  |
|  | Target group: Is the app content (visual information, language, design) appropriate for your target audience? | 1 Completely inappropriate/ unclear/confusing 2 Mostly inappropriate/unclear/ confusing 3 Acceptable but not targeted. May be inappropriate/unclear/ confusing 4 Well-targeted, with negligible issues 5 Perfectly targeted, no issues found |  |

**Supplementary Table 1.** Assessment criteria of the apps (continued).

| Functionality – app functioning, easy to learn, navigation, flow logic, and gestural design of app | Performance: How accurately/fast do the app features (functions) and components (buttons/menus) work? | 1 App is broken; no/insufficient/ inaccurate response (e.g., crashes/bugs/broken features, etc.) 2 Some functions work, but lagging or contains major technical problems 3 App works overall. Some technical problems need fixing/Slow at times 4 Mostly functional with minor/negligible problems 5 Perfect/timely response; no technical bugs found/contains a ‘loading time left’ indicator |  |
| --- | --- | --- | --- |
|  | Ease of use: How easy is it to learn how to use the app; how clear are the menu labels/icons and instructions? | 1 No/limited instructions; menu labels/icons are confusing; complicated 2 Useable after a lot of time/effort 3 Useable after some time/effort 4 Easy to learn how to use the app (or has clear instructions) 5 Able to use app immediately; intuitive; simple |  |
|  | Navigation: Is moving between screens logical/accurate/ appropriate/ uninterrupted; are all necessary screen links present? | 1 Different sections within the app seem logically disconnected and random/confusing/ navigation is difficult 2 Usable after a lot of time/effort 3 Usable after some time/effort 4 Easy to use or missing a negligible link 5 Perfectly logical, easy, clear and intuitive screen flow throughout, or offers shortcuts |  |
|  | Gestural design: Are interactions (taps/swipes/pinches/ scrolls) consistent and intuitive across all components/screens? | 1 Completely inconsistent/ confusing 2 Often inconsistent/confusing 3 OK with some inconsistencies/confusing elements 4 Mostly consistent/intuitive with negligible problems 5 Perfectly consistent and intuitive |  |
| Aesthetics – graphic design, overall visual appeal, colour scheme, and stylistic consistency | Layout: Is arrangement and size of buttons/icons/ menus/content on the screen appropriate or zoomable if needed? | 1 Very bad design, cluttered, some options impossible to select/locate/see/read device display not optimised 2 Bad design, random, unclear, some options difficult to select/locate/see/read 3 Satisfactory, few problems with selecting/locating/ seeing/reading items or with minor screen size problems 4 Mostly clear, able to select/ locate/see/read items 5 Professional, simple, clear, orderly, logically organised, device display optimised. Every design component has a purpose |  |

**Supplementary Table 1.** Assessment criteria of the apps (continued).

|  | Graphics: How high is the quality/resolution of graphics used for buttons/icons/menus/ content? | 1 Graphics appear amateur, very poor visual design - disproportionate, completely stylistically inconsistent 2 Low quality/low resolution graphics; low quality visual design – disproportionate, stylistically inconsistent 3 Moderate quality graphics and visual design (generally consistent in style) 4 High quality/resolution graphics and visual design – mostly proportionate, stylistically consistent 5 Very high quality/resolution graphics and visual design - proportionate, stylistically consistent throughout |  |
| --- | --- | --- | --- |
|  | Visual appeal: How good does the app look? | 1 No visual appeal, unpleasant to look at, poorly designed, clashing/mismatched colours 2 Little visual appeal – poorly designed, bad use of colour, visually boring 3 Some visual appeal – average, neither pleasant, nor unpleasant 4 High level of visual appeal – seamless graphics – consistent and professionally designed 5 As above + very attractive, memorable, stands out; use of colour enhances app features/menus |  |
| Information – Contains high quality information (e.g., text, feedback, measures, references) from a credible source. Select N/A if the app component is irrelevant. | Accuracy of app description (in app store): Does app contain what is described? | 1 Misleading. App does not contain the described components/functions. Or has no description 2 Inaccurate. App contains very few of the described components/functions 3 OK. App contains some of the described components/functions 4 Accurate. App contains most of the described components/ functions 5 Highly accurate description of the app components/functions |  |
|  | Goals: Does app have specific, measurable, and achievable goals (specified in app store description or within the app itself)? | N/A Description does not list goals, or app goals are irrelevant to research goal (e.g., using a game for educational purposes) 1 App has no chance of achieving its stated goals 2 Description lists some goals, but app has very little chance of achieving them 3 OK. App has clear goals, which may be achievable. 4 App has clearly specified goals, which are measurable and achievable 5 App has specific and measurable goals, which are highly likely to be achieved |  |

**Supplementary Table 1.** Assessment criteria of the apps (continued).

|  | Quality of information: Is app content correct, well written, and relevant to the goal/topic of the app? | N/A There is no information within the app 1 Irrelevant/inappropriate/ incoherent/incorrect 2 Poor. Barely relevant/ appropriate/ coherent/may be incorrect 3 Moderately relevant/ appropriate/coherent/ and appears correct 4 Relevant/appropriate/coherent /correct 5 Highly relevant, appropriate, coherent, and correct |  |
| --- | --- | --- | --- |
|  | Quantity of information: Is the extent coverage within the scope of the app; and comprehensive but concise? | N/A There is no information within the app 1 Minimal or overwhelming 2 Insufficient or possibly overwhelming 3 OK but not comprehensive or concise 4 Offers a broad range of information, has some gaps or unnecessary detail; or has no links to more information and resources 5 Comprehensive and concise; contains links to more information and resources |  |
|  | Visual information: Is visual explanation of concepts – through charts/graphs/images/ videos, etc. – clear, logical, correct? | N/A There is no visual information within the app (e.g., it only contains audio, or text) 1 Completely unclear/confusing/ wrong or necessary but missing 2 Mostly unclear/confusing/ wrong 3 OK but often unclear/ confusing/wrong 4 Mostly clear/logical/correct with negligible issues 5 Perfectly clear/logical/correct |  |
|  | Credibility: Does the app come from a legitimate source (specified in app store description or within the app itself)? | 1 Source identified but legitimacy/trustworthiness of source is questionable (e.g. commercial business with vested interest) 2 Appears to come from a legitimate source, but it cannot be verified (e.g. has no webpage) 3 Developed by small NGO /institution (hospital/centre, etc.) /specialised commercial business, funding body 4 Developed by government, university or as above but larger in scale 5 Developed using nationally competitive government or research funding (e.g. Australian Research Council, NHMRC) |  |

**Supplementary Table 1.** Assessment criteria of the apps (continued).

|  | Evidence base: Has the app been trialled/tested; must be verified by evidence (in published scientific literature)? | N/A The app has not been trialled/tested 1 The evidence suggests the app does not work 2 App has been trialled (e.g., acceptability, usability, satisfaction ratings) and has partially positive outcomes in studies that are not randomised controlled trials (RCTs), or there is little or no contradictory evidence. 3 App has been trialled (e.g., acceptability, usability, satisfaction ratings) and has positive outcomes in studies that are not RCTs, and there is no contradictory evidence. 4 App has been trialled and outcome tested in 1-2 RCTs indicating positive results 5 App has been trialled and outcome tested in > 3 high quality RCTs indicating positive results |  |
| --- | --- | --- | --- |
|  | Please record your overall impressions of this app and any other observations from this app testing. | Free text | To record any significant but missing information. |
| **MARS App Subjective Quality** | | |  |
|  | Would you recommend this app to people who might benefit from it? | 1 Not at all I would not recommend this app to anyone 2 There are very few people I would recommend this app to 3 Maybe There are several people whom I would recommend it to 4 There are many people I would recommend this app to 5 Definitely I would recommend this app to everyone |  |
|  | How many times do you think you would use this app in the next 12 months if it was relevant to you? | 1 None 2 1-2 3 3-10 4 10-50 5 >50 |  |
|  | Would you pay for this app? | 1 No 3 Maybe 5 Yes |  |
|  | What is your overall star rating of the app? | 1 « One of the worst apps I’ve used 2 «« 3 ««« Average 4 «««« 5 ««««« One of the best apps I've used |  |

**Supplementary Table 1.** Assessment criteria of the apps (continued).

| **mHON** |  |  |  |
| --- | --- | --- | --- |
| Authority | Does the app indicate the qualifications of specific individuals who developed the app or contributed to the information provided? | Yes/no | The qualifications of the authors are indicated. |
| Complementarity | Is there a disclaimer stating or which implies that the information provided and/or app functions do not replace the healthcare provider's advice? | Yes/No/Advice given by healthcare provider | Information should support, not replace, the doctor-patient relationship. |
| Confidentiality | Is there a privacy and confidentiality clause in the app? | Yes/No |  |
|  | Is the privacy policy easily accessible within the app? | Yes/No |  |
|  | Is consent to data collection required at the first launch of the application? | yes/no |  |
|  | Where the data is stored (on the application, on the SD card, etc.) | Free text |  |
|  | Is the data transmitted to third parties? | yes/no |  |
|  | If yes, to which third parties are they transmitted? |  |  |
|  | Is the privacy policy clear about data sharing and with whom? | yes/no |  |
| Validity | Is the date of last general update provided? | Yes/No | The user must be able to easily know how current the health information is. And the legal content (Legal Notice; Terms and Conditions; Privacy Policy) should have a date of last update. |
| Justifiability | Does the health information have references, is it complete and provided in an objective manner? | **Yes** (claims are backed up by evidence)/ **Inconsistent**/**No** (claims not backed up by evidence)/ **NA** (no claims) |  |
| User's practice | Is the target audience clearly stated? (e.g., Patients, health professionals etc.) | Yes/No (elaborate on audience) |  |
|  | If the application is prohibited for use by minors, is it clearly indicated and designed in a way that use by minors is impossible? | Yes/No (how?) |  |
|  | Are the developers contactable by email? (The email may be found in the app, the app store, app developer's website, etc) | Yes/No |  |
|  | Are there instructions provided so that the application is easy to use without any bugs? | Yes/No |  |
| Financial disclosure | Does the app indicate any funding sources? | **Yes** (the app is managed by a registered commercial company)  **No** (the app is managed by an individual developer without funding information) | Identify funding sources. |
|  | If yes, which body created/ commissioned/ partnered the app? | Free text |  |
|  | If no, who created/ commissioned/ partnered the app? | Free text |  |
| Advertising Policy | Are advertorials distinguishable from content of the app? | Yes/No/No advertising | Clearly distinguish advertising from editorial content. |
|  | Do the adverts interfere with the functioning of the app (if any?) | Yes/No/No adverts | Desirable not to have adverts as it may prevent the proper functioning of the app. |
| Other Remarks | Any remarks from reviewer? | Free Text |  |
