# Supplementary material for: Scope, Characteristics, Behavior Change Techniques, and Quality of Conversational Agents for Mental Health and Well-Being: Systematic Assessment of Apps
Source: J Med Internet Res. 2023 Jul 18;25:e45984. doi: 10.2196/45984 (PMC10394504; doi:10.2196/45984)
Supplement: Multimedia Appendix 2 [file jmir_v25i1e45984_app2.docx]

**Multimedia Appendix 2. Search terms**

*AI companion, AI buddy, AI coach, health friend, health buddy, health chat companion, chatbot, AI chatbot, stress chatbot, mental health chatbot, artificial intelligent chatbot, artificial intelligence chatbot, AI friend, therapy chatbot, bot, wellness chatbot, wellbeing chatbot, mental health companion, conversational agent, depression chatbot, anxiety chatbot, relaxation chatbot, mindfulness chatbot,* and *self-care chatbot*.
