# Supplementary figures and images for: Scope, Characteristics, Behavior Change Techniques, and Quality of Conversational Agents for Mental Health and Well-Being: Systematic Assessment of Apps
Source: J Med Internet Res. 2023 Jul 18;25:e45984. doi: 10.2196/45984 (PMC10394504; doi:10.2196/45984)

**Multimedia Appendix 3.** Word Cloud of App Descriptions.


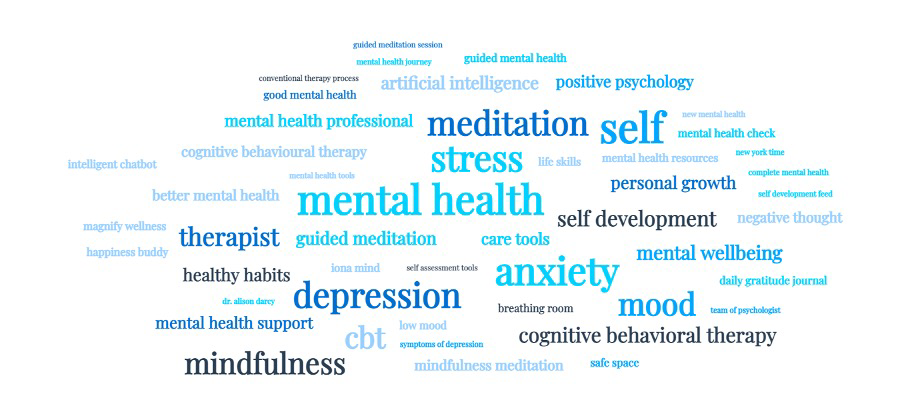

Supplement: Multimedia Appendix 3 [file jmir_v25i1e45984_app3.docx]
