# Supplementary material for: Scope, Characteristics, Behavior Change Techniques, and Quality of Conversational Agents for Mental Health and Well-Being: Systematic Assessment of Apps
Source: J Med Internet Res. 2023 Jul 18;25:e45984. doi: 10.2196/45984 (PMC10394504; doi:10.2196/45984)
Supplement: Multimedia Appendix 4 [file jmir_v25i1e45984_app4.docx]

**Multimedia Appendix 4**. Characteristics of included apps.

| App characteristics | | | | App functionalities | | | | | | | |
| --- | --- | --- | --- | --- | --- | --- | --- | --- | --- | --- | --- |
| App Name | Platform | App category | Developed by | Information and Education | | Evidence | | Human involvement | | Emergency safety netting | |
|  |  |  |  | Has psycho-educat-ion | Provides specific information on CMDs | Includes references | Evaluated in studies | Involves user's support network | Provides direct access to health profession-als | Screens risk of suicide | Can contact emergency services |
| Woebot | Android, iOS | Medical, Health & Fitness | Woebot Health or Woebot Labs Inc | Yes | No | Yes | Yes | No | No | No (but has SOS button which triggers emergency response) | Yes (provides emergency number for USA, Canada and EU) |
| Jumping Minds | Android | Health & Fitness | Jumping Minds | No | No | No | No | Yes (connects with peers) | No | No | No |
| Lissun | Android | Health & Fitness | Mindeye Solutions Pvt Ltd | Yes | No | No | No | No | Yes | No | No |
| Talk to Poppy | Android | Health & Fitness | PromethistAI a.s. | Yes | No | No | No | No | No | Yes (responds to suicide-related keywords, e.g. "suicidal thoughts") | Yes (provides emergency numbers specific to location) |
| GritX | Android, iOS | Health & Fitness | WSC Technology, Inc. | Yes | Yes | Yes | No | No | No | Yes (responds to suicide-related keywords, e.g. "suicide") | Yes (provides emergency numbers specific to location) |
| Inner-Hour or Amaha* | Android, iOS | Health & Fitness | InnerHour | Yes | Yes | Yes, some linked articles | No | Yes | Yes | No | No |
| Iona | Android, iOS | Health & Fitness | Iona Mind - Mental Health Support | Yes | No | Yes | No | No | No | No | Yes (provides list of emergency numbers for many locations) |
| IWill Care | Android, iOS | Health & Fitness, Medical | IWIll; Svieda Ventures Private Limited, Epsyclinic | Yes | No | No | No | No | Yes | No | Yes (hotline for specific location) |
| Mindspa | Android | Health & Fitness | Mindspa Health | Yes | No | No | No | No | No | No | No |
| Nuna | Android, iOS | Lifestyle | Nuna, Nuna Technologies ApS | Yes | Yes | Yes | Studies in progress | No | No | No | No |
| tomo | Android, iOS | Health & Fitness | ICAS Digital Health Ltd | No | No | No | Studies in progress | No | No | No | No (but provides a link to website with mental health resources and emergency contact details) |
| Wysa | Android, iOS | Health & Fitness | Touchkin, Touchkin eServices Private Limited | Yes | No | Yes | Yes | No | No | No (but has SOS button which triggers emergency response) | Yes |
| Zifcare | Android | Health & Fitness | Zifcare Technologies | Yes | Yes | No | No | No | No | No | No |
| Happify | Android, iOS | Health & Fitness | Happify, Inc. | Yes | No | Yes | Yes | Yes | No | No | No |
| Aiki | iOS | Health & Fitness | Cognitive Apps Software Solutions Inc. | Yes | Yes | No | No | No | No | No | No |
| Inwords | iOS | Lifestyle | Symbiose Technologies | Yes (referral to articles) | Yes (referral to articles) | Yes, some linked articles | No | No | No | No | No |
| Magnify Wellness | Android, iOS | Health & Fitness | Abigayle Peterson | Yes | No | No | No | No | No | No | No |
| I’m Fine | iOS | Health & Fitness | Ascendio TechVision | Yes | Yes | No | No | No | Yes | No | No |
| *“InnerHour: self-care therapy” has been renamed to “Amaha: anxiety sleep self care”. In this paper, we still refer to it as “InnerHour”, as our team coded the content in the app while it was still called InnerHour. | | | | | | | | | | | |
