# Supplementary material for: Scope, Characteristics, Behavior Change Techniques, and Quality of Conversational Agents for Mental Health and Well-Being: Systematic Assessment of Apps
Source: J Med Internet Res. 2023 Jul 18;25:e45984. doi: 10.2196/45984 (PMC10394504; doi:10.2196/45984)
Supplement: Multimedia Appendix 5 [file jmir_v25i1e45984_app5.docx]

**Multimedia Appendix 5**. Other characteristics of included apps (N=18).

| Characteristic | | Value |
| --- | --- | --- |
| **Affiliation, n (%)** | |  |
|  | Commercial | 16 (89) |
|  | University | 1 (6) |
|  | Unknown | 1 (6) |
| **Relevant Expertise Involved, n (%)** | |  |
|  | No | 2 (11) |
|  | Not specified | 4 (22) |
|  | Yes^a^ | 12 (67) |
| **Technical Aspects*, n (%)** | |  |
|  | Needs internet access to function | 18 (100) |
|  | Sends reminders | 14 (78) |
|  | Requires login | 12 (67) |
|  | Includes customizable user profile | 5 (28) |
|  | Has an app community | 4 (22) |
|  | Provides additional security protection^b^ | 2 (11) |
|  | Allows exporting of data | 1 (6) |
|  | Allows sharing (Instagram) | 1 (6) |
|  | Uses phone sensors | 1 (6) |
| **CA Integration, n (%)** | |  |
|  | Part of a multifunctional app | 13 (72) |
|  | Standalone chatbot | 5 (28) |
| **Has Default Message^c^, n (%)** | |  |
|  | Yes | 7 (39) |
|  | No | 11 (61) |
| **Personalisation*, n (%)** | |  |
|  | Personalised timing | 10 (56) |
|  | Keeps history of user preferences | 9 (50) |
|  | Remembers previous conversations | 9 (50) |
|  | Records users’ health status | 6 (33) |
|  | Saves users’ goals | 4 (22) |
| **Evidence base, n (%)** | |  |
|  | Evaluation studies | 3 (17) |
|  | Design/development studies | 2 (11) |
|  | No studies | 13 (72) |
| **Human support*, n (%)** | |  |
|  | Peer-support | 3 (17) |
|  | Health professionals | 4 (22) |
|  | No human support | 12 (67) |
| **Emergency safety netting*, n (%)** | |  |
|  | Screens risk of suicide | 2 (11) |
|  | Can contact emergency services | 6 (33) |
|  | No emergency safety netting | 12 (67) |
| **Other functionalities*, n (%)** | |  |
|  | Journaling | 9 (50) |
|  | Mood monitoring | 7 (39) |
|  | Gratitude | 5 (28) |
|  | Games | 2 (11) |
| Note: Some apps (*) may present with more than 1 feature within the sections, therefore some sections do not add up to 100%.  ^a^Includes medical doctors, psychiatrists, neuroscientists, psychologists, or other mental health professionals in the development team ^b^Provides option for biometric lock and security pin  ^c^Having a default message in response to not understanding the user input. | | |
