# Supplementary material for: Scope, Characteristics, Behavior Change Techniques, and Quality of Conversational Agents for Mental Health and Well-Being: Systematic Assessment of Apps
Source: J Med Internet Res. 2023 Jul 18;25:e45984. doi: 10.2196/45984 (PMC10394504; doi:10.2196/45984)
Supplement: Multimedia Appendix 6 [file jmir_v25i1e45984_app6.docx]

**Multimedia Appendix 6**. Number of BCTs found in each app (N=18).

| Characteristic | | BCTs |
| --- | --- | --- |
| Number of BCTs, mean (SD) | | 15.8 (8.77) |
| **Number of BCTs per app** | |  |
|  | Woebot | 30 |
|  | Wysa: Anxiety, therapy chatbot | 29 |
|  | Happify | 25 |
|  | Inwords: Reflect with Remy | 25 |
|  | Nuna: Mental Health Companion | 23 |
|  | Aiki - stress test & self care | 23 |
|  | Talk to Poppy: AI friend | 20 |
|  | Iona: Mental Health Journal, Therapy & Anxiety App | 20 |
|  | I’m Fine: Mental health Guide | 16 |
|  | Lissun | 14 |
|  | InnerHour: self-care therapy | 13 |
|  | tomo | 10 |
|  | IWill Care | 10 |
|  | Jumping Minds - Feel Better | 6 |
|  | GritX | 6 |
|  | Mindspa: The Mental Health App | 6 |
|  | Magnify Wellness | 5 |
|  | Zifcare Self Development App | 4 |
