# Supplementary material for: Scope, Characteristics, Behavior Change Techniques, and Quality of Conversational Agents for Mental Health and Well-Being: Systematic Assessment of Apps
Source: J Med Internet Res. 2023 Jul 18;25:e45984. doi: 10.2196/45984 (PMC10394504; doi:10.2196/45984)
Supplement: Multimedia Appendix 8 [file jmir_v25i1e45984_app8.docx]

**Multimedia Appendix 8.** Common BCTs by level of CA intelligence, mental health topics, interventions, and therapeutic components.

|  | BCT | Subgroup |  |  |  |  | Implementation |
| --- | --- | --- | --- | --- | --- | --- | --- |
| **CA Intelligence, n (%)** | | Rule-based | AI-enhanced |  |  |  |  |
|  | *1.4 “Action planning”* | NA | 7 (78) |  |  |  | Plans actionable steps and timings to carry out the task |
|  | 3.3 “Social support (emotional)” | 7 (78) | 8 (89) |  |  |  | "I'm sorry to hear you are feeling sad. Here, take a hug from me [GIF of CA]" [InnerHour] |
|  | 4.1 “Instructions for how to perform a behaviour” | 7 (78) | 9 (100) |  |  |  | "First thing, make a list of whatever it is you need to accomplish by the end of the day. Then prioritise the list, according to importance. After completion of each task, cross it off and eliminate it from your list" [Inwords] |
|  | *5.6 “Information about emotional consequences”* | NA | 8 (89) |  |  |  | Given to encourage participation in the suggested activity, e.g., "This can help interrupt our negative thoughts and boost our experiences of pleasure, achievement, and mastery which can help change our mood" [Nuna] |
|  | 6.1 “Demonstration of the behaviour” | NA | 8 (89) |  |  |  | Describes one CA as someone with similar experiences of negative thoughts and how she overcame them [Talk to Poppy] |
|  | *7.1 “Prompts/cues”* | NA | 8 (89) |  |  |  | Provides reminders and notifications |
|  | *10.4 “Social reward”* | NA | 7 (78) |  |  |  | Congratulates user on improvement, e.g. "This is really good going, and we've made some great progress today" [Iona] |
|  | 11.2 “Reduce negative emotions” | NA | 9 (100) |  |  |  | "Imagine yourself on some island where you feel safe and sound… Not only will this shift your mood, but it can also make you more productive at work when you get back to it." [Aiki] |
|  | *12.6 “Body changes”* | NA | 9 (100) |  |  |  | Relaxation, breathing, meditation, and stretching exercises |
|  | *13.2 “Framing/ reframing”* | NA | 7 (78) |  |  |  | Guides user through a cognitive restructuring exercise, e.g. "We'll try to rethink this thought: [I don’t have the energy to do anything]. Remember our thoughts are not always helpful." [Wysa] |
| **Mental Health Topic, n (%)** | | Wellbeing | Stress | Anxiety | Depression |  |  |
|  | 3.3 “Social support (emotional)” | 15 (83) | 12 (80) | 11 (85) | 9 (75) |  | "Great, I'm glad you were able to complete the activity." [InnerHour] |
|  | 4.1 “Instructions for how to perform a behaviour” | 16 (89) | 13 (87) | 12 (92) | 11 (92) |  | "Write down your negative thoughts, process them, and then, watch them fade away" [Jumping Minds] |
|  | *5.6 “Information about emotional consequences”* | NA | NA | NA | 9 (75) |  | "Writing about your feelings can help you feel calmer… The act of writing is a cathartic process in itself. It can help release pent-up feelings" [InnerHour] |
|  | 6.1 “Demonstration of the behaviour” | 14 (78) | 12 (80) | 10 (77) | 10 (83) |  | Provide video of breathing exercise count, and progressive muscle relaxation [Woebot] |
|  | 11.2 “Reduce negative emotions” | 15 (83) | 13 (87) | 12 (92) | 11 (92) |  | “This technique (gives us an overview of the whole situation...understanding that we do not carry all the responsibility) reduces the feeling of sadness” [I’m Fine] |
|  | *12.6 “Body changes”* | NA | NA | 10 (77) | 9 (75) |  | Relaxation, breathing, stretching exercises |
| **Intervention, n (%)** | | CBT | Mindfulness | Positive Psychology | DBT |  |  |
|  | 3.3 “Social support (emotional)” | 12 (86) | 9 (90) | 4 (100) | 3 (100) |  | "Oh no! I am here to listen to you. This is a safe space where you can freely vent your emotions. What is causing you to feel your emotions? If you want to rant to me, enter your response below!" [Magnify Wellness] |
|  | 4.1 “Instructions for how to perform a behaviour” | 14 (100) | 9 (90.0) | 4 (100) | 3 (100) |  | "Set aside at least 10 minutes for your savoring experience and try to limit any distractions while you’re doing it… Pay attention to all of your senses and to the emotions you feel during the experience" [Happify] |
|  | *5.1 “Information about*  *health consequences”* | NA | NA | 3 (75) | NA |  | "When we feel tired, tension starts to build up in our muscles. A good stretch can help release all that tension. It can also improve blood flow and give you a burst of much-needed energy" [InnerHour] |
|  | *5.4 “Monitoring of emotional consequences”* | NA | NA | 3 (75) | NA |  | "How do you feel now?" after activity [Lissun] |
|  | *5.6 “Information about emotional consequences”* | 11 (79) | 8 (80) | 3 (75) | NA |  | Provides information on the benefits of suggested tasks, e.g. “The good thing is, if we find a way to think differently about the same situation or event, it may help us feel a lot better.” [IWill Care] |
|  | 6.1 “Demonstration of the behaviour” | 12 (86) | 8 (80) | 4 (100) | NA |  | "As for me, from "I am a failure"-I changed it to "I am working hard. I will be a good bot as I keep learning" [IWill Care] |
|  | *8.1 “Behavioural practice/rehearsal”* | NA | NA | 3(75) | 3 (100) |  | Mindfulness practice for reducing anxiety [Wysa] |
|  | 11.2 “Reduce negative emotions” | 13 (93) | 9 (90) | 3(75) | 3 (100) |  | Give poems and inspirational quotes "Sometimes it takes only one act of kindness to change a person's life - Jackie Chan" [Zifcare] |
|  | *12.4 “Distraction”* | NA | NA | 3 (75) | NA |  | Plays a game where user has to click on the hot air balloons with positive words [Happify] |
|  | *12.6 “Body changes”* | 12 (86) | 8 (80) | 3 (75) | 3 (100) |  | 10-minute physical exercise video [Magnify Wellness] |
| **Therapeutic component, n (%)** | | Psychoeducation | Relaxation | Problem solving | Behavioural activation | Cognitive restructuring |  |
|  | *1.2 “Problem solving”* | NA | NA | 9 (90) | 7 (78) | NA | "Let's take a close look at your problem, I can help you solve it... is the description you just wrote specific? ... Now that you have a solid problem description, let's come up with a problem-solving goal... I'd like you to write down 5-7 ideas on how you might achieve your problem-solving goal” [Woebot] |
|  | *1.4 “Action planning”* | NA | NA | 8 (80) | 8 (89) | 8 (89) | "Let's think of some ways and some small steps that you could do today to tackle this, one small step that could move things in the direction you'd want. What, or who could help you with that at this moment?" [Talk to Poppy] |
|  | 3.3 “Social support (emotional)” | 14 (88) | 12 (92) | NA | NA | 8 (89) | "I understand that you feel depressed and want to help you."[GritX] |
|  | 4.1 “Instructions for how to perform a behaviour” | 14 (88) | 13 (100) | 10 (100) | 9 (100) | 9 (100) | For mindful walking task: "As you start to walk, observe what is happening around you. Try not to focus on the thoughts in your mind. Instead, focus on what you can see or hear. Then, focus on your other sense as well - what you can feel, smell, and taste." [InnerHour] |
|  | *5.6 “Information about emotional consequences”* | 12 (75.0) | 11 (85) | 9 (90) | 8 (89) | 9 (100) | "Keeping a gratitude journal can help boost your positive emotions when you experience them and make you feel better when things are not going well" [Iona] |
|  | 6.1 “Demonstration of the behaviour” | 14 (88) | 12 (92) | 9 (90) | 8 (89) | 9 (100) | "In this moment, after I make my plan, I can do some self-care to help my body feel calmer and safer. I could take a bath, take a walk, clean my home, treat myself to a hot meal, or do some exercises from Mindspa" [Mindspa] |
|  | *7.1 “Prompts/cues”* | NA | NA | 8 (80) | 8 (89) | 7 (78) | uses notifications to remind you to do the tasks you wanted to make a habit |
|  | *10.4 “Social reward”* | NA | NA | 9 (90) | 8 (89) | 8 (89) | “Thank you for coming back! I'm pleased to speak with you again” [Talk to Poppy] |
|  | 11.2 “Reduce negative emotions” | 15 (94) | 13 (100) | 9 (90) | 8 (89) | 9 (100) | “Mindfulness is another great way to manage worry.” [Wysa] |
|  | *12.6 “Body changes”* | 13 (81) | 13 (100) | 8 (80) | 7 (78) | 8 (89) | Relaxation, breathing, stretching exercises, etc. |
|  | *13.2 “Framing/ reframing”* | NA | NA | 8 (80) | NA | 9 (100) | "Now let's look back through what you've written down, and use it to help you rewrite your thought", "Let's move on to looking for evidence against your unhelpful thought" [Iona] |
| Note: Additional BCTs that were not common across all apps are italicised. | | | | | | | |
