# Supplementary material for: Scope, Characteristics, Behavior Change Techniques, and Quality of Conversational Agents for Mental Health and Well-Being: Systematic Assessment of Apps
Source: J Med Internet Res. 2023 Jul 18;25:e45984. doi: 10.2196/45984 (PMC10394504; doi:10.2196/45984)
Supplement: Multimedia Appendix 9 [file jmir_v25i1e45984_app9.docx]

**Multimedia Appendix 9.** Health on the Net Foundation certification (mHONcode) characteristics of the included apps (N=18).

| Characteristic | | | Value | |
| --- | --- | --- | --- | --- |
| **Authority** | | | | |
| Qualifications of app developers indicated, n (%) | | | | |
|  | Yes | | 12 | (67) |
|  | Yes (not healthcare professionals) | | 2 | (11) |
|  | No | | 4 | (22) |
| **Complementarity** | | | | |
| Disclaimer that app does not replace the healthcare provider's advice, n (%) | | |  | |
|  | Yes | | 17 | (94) |
|  | No | | 1 | (6) |
| **Confidentiality** | | | | |
| Privacy clause on app, n (%) | | | | |
|  | Yes | | 17 | (94) |
|  | No | | 1 | (6) |
| Accessible privacy policy within app, n (%) | | |  |  |
|  | Yes | | 14 | (78) |
|  | No | | 4 | (22) |
| Requested for consent to data collection, n (%) | | | | |
|  | Yes | | 2 | (11) |
|  | No | | 16 | (89) |
| Data sent to third parties, n (%) | |  | | |
|  | Yes | | 17 | (94) |
|  | No | | 1 | (6) |
| Clear data sharing policy, n (%) | |  | | |
|  | Yes | | 7 | (39) |
|  | No | | 5 | (28) |
|  | Not specific | | 6 | (33) |
| **Validity** | | | | |
| Date of last general update provided, n (%) | | | | |
|  | Yes | | 18 | (100) |
|  | No | | 0 | (0) |
| **Justifiability** | | | | |
| Health references included in objective manner, n (%) | | | | |
|  | Yes | | 3 | (17) |
|  | No | | 9 | (50) |
|  | inconsistent | | 6 | (33) |
| **User’s Practice** | | | | |
| Target audience clearly stated, n (%) | | | | |
|  | Yes | | 18 | (100) |
|  | No | | 0 | (0) |
| Prohibits use of app by minors, n (%) | |  | | |
|  | Requires user acknowledgement | | 2 | (11) |
|  | No | | 16 | (89) |
| Developers contactable by email, n (%) | | | | |
|  | Yes | | 16 | (89) |
|  | No | | 2 | (11) |
| App instructions included or easy to use, n (%) | | | | |
|  | Yes | | 18 | (100) |
|  | no | | 0 | (0) |
| **Financial Disclosure** | |  | | |
| Funding sources indicated, n (%) | |  | | |
|  | Yes | | 16 | (89) |
|  | No | | 2 | (11) |
| **Advertising Policy** | | |  |  |
| Advertorials distinguishable from content of the app, n (%) | | | | |
|  | No advertising | | 18 | (100) |
