# Supplementary material for: Scope, Characteristics, Behavior Change Techniques, and Quality of Conversational Agents for Mental Health and Well-Being: Systematic Assessment of Apps
Source: J Med Internet Res. 2023 Jul 18;25:e45984. doi: 10.2196/45984 (PMC10394504; doi:10.2196/45984)
Supplement: Multimedia Appendix 10 [file jmir_v25i1e45984_app10.docx]

**Multimedia Appendix 10.** Ratings of included apps (N=18).

| App Name | MARS Score |  |  |  |  |  | Consumer Ratings |  |
| --- | --- | --- | --- | --- | --- | --- | --- | --- |
|  | Engagement | Functionality | Aesthetics | Information | Total Quality | Subjective Quality | Average User Ratings | Total number of ratings |
| Woebot | 4.10 | 4.75 | 4.67 | 4.33 | 4.46 | 3.88 | 4.75 | 16890 |
| Jumping Minds | 2.70 | 4.25 | 4.33 | 3.08 | 3.59 | 2.50 | 3.7 | 1809 |
| Lissun | 2.80 | 4.25 | 4.33 | 3.80 | 3.80 | 2.63 | 4.67 | 15 |
| Talk to Poppy | 3.10 | 3.88 | 3.83 | 3.88 | 3.67 | 2.38 | 3.2 | 844 |
| GritX | 3.30 | 3.63 | 4.00 | 3.83 | 3.69 | 2.25 | 3.75 | 72 |
| InnerHour | 3.80 | 4.13 | 4.17 | 3.60 | 3.92 | 3.25 | 4.25 | 23665 |
| Iona | 3.90 | 4.63 | 4.33 | 3.38 | 4.06 | 3.50 | 4.65 | 880 |
| IWill Care | 3.00 | 3.50 | 4.00 | 3.20 | 3.43 | 2.25 | 2.04 | 10 |
| Mindspa | 2.40 | 3.00 | 3.67 | 3.00 | 3.02 | 2.25 | 4.05 | ‎2899 |
| Nuna | 4.00 | 4.25 | 4.00 | 3.67 | 3.98 | 3.50 | 4 | 145 |
| tomo | 2.90 | 4.38 | 4.33 | 3.50 | 3.78 | 2.50 | 5 | 21 |
| Wysa | 4.50 | 4.50 | 4.50 | 4.43 | 4.48 | 4.38 | 4.8 | 120886 |
| Zifcare | 2.40 | 4.25 | 3.33 | 3.25 | 3.31 | 2.00 | 4.7 | 913 |
| Happify | 4.30 | 4.25 | 4.33 | 3.93 | 4.20 | 4.00 | 4.2 | 7112 |
| Aiki | 3.50 | 4.63 | 4.50 | 2.58 | 3.80 | 2.88 | 4.7 | 25 |
| Inwords | 3.80 | 4.75 | 4.50 | 3.75 | 4.20 | 2.88 | 4.4 | 329 |
| Magnify Wellness | 2.60 | 3.63 | 2.83 | 2.67 | 2.93 | 1.38 | 4.7 | 70 |
| I’m Fine | 2.90 | 4.13 | 4.50 | 4.00 | 3.88 | 2.88 | 4.86 | 22 |
| Note: The MARS Total Quality score was calculated as the average of Engagement, Functionality, Aesthetics, and Information quality subscales. The Subjective Quality score was excluded from the Total Quality score as it is based on an individual’s subjective assessment of the apps, which in line with the MARS development paper by Stoyanov et al. (2015). | | | | | | | | |
